# Th1/Th2 Cells and Associated Cytokines in Acute Hepatitis E and Related Acute Liver Failure

**DOI:** 10.1155/2020/6027361

**Published:** 2020-11-17

**Authors:** Jian Wu, Yurong Guo, Xuan Lu, Fen Huang, Feifei Lv, Daqiao Wei, Anquan Shang, Jinfeng Yang, Qiaoling Pan, Bin Jiang, Jiong Yu, Hongcui Cao, Lanjuan Li

**Affiliations:** ^1^State Key Laboratory for Diagnosis and Treatment of Infectious Diseases, National Clinical Research Center for Infectious Diseases, The First Affiliated Hospital, Zhejiang University School of Medicine, 79 Qingchun Rd., Hangzhou 310003, China; ^2^Department of Laboratory Medicine, Yancheng Clinical Medical College of Nanjing Medical University, Yancheng 224001, China; ^3^Department of Laboratory Medicine, Yancheng Hospital of Traditional Chinese Medicine, Affiliated to Nanjing University of Traditional Chinese Medicine, Yancheng 224000, China; ^4^Medical School, Kunming University of Science and Technology, 727 Jing Ming South Road, Kunming 650031, China; ^5^Department of Laboratory Medicine, The First Affiliated Hospital, College of Medicine, Zhejiang University, Hangzhou, China; ^6^Department of Clinical Laboratory, Shanghai Tongji Hospital, Tongji University School of Medicine, 389 Xincun Road, Shanghai 200065, China; ^7^Department of Laboratory Medicine, The Central Blood Station of Yancheng City, Yancheng, 224000 Jiangsu, China; ^8^Zhejiang Provincial Key Laboratory for Diagnosis and Treatment of Aging and Physic-chemical Injury Diseases, 79 Qingchun Rd, Hangzhou 310003, China

## Abstract

**Background and Aims:**

The involvement of cellular immunity in the development of hepatitis E virus (HEV) infection is rare. We aimed to study the roles of viral load and Th cell responses in acute hepatitis E (AHE) and HEV-related acute liver failure (HEV-ALF).

**Methods:**

We evaluated viral load and Th1/Th2 cytokine levels in 34 patients with HEV infection, including 17 each with AHE or HEV-ALF. Seventeen healthy controls (HCs) were also included who were negative for anti-HEV IgM and IgG.

**Results:**

There was no significant difference in viral load and HEV RNA in the AHE and HEV-ALF groups (both *P* > 0.05). The Th lymphocyte levels (CD3+, CD4+) in the AHE and HEV-ALF groups were significantly higher than those in the HC group (both *P* < 0.05), but there was no significant difference between the AHE and HEV-ALF groups (*P* > 0.05). Both IFN-*γ* and IL-10 showed gradual upward trend from the HC group to the AHE (both *P* < 0.01), but IFN-*γ* showed a sharp downward trend from the AHE group to the HEV-ALF group (*P* < 0.01) and IL-4 showed gradual upward trend from the AHE group to the HEV-ALF group (*P* < 0.01).There was no significant difference in Th1 and Th2 cytokines between the HEV RNA(+) group and HEV RNA(-) group (all *P* > 0.05). Th2 bias was observed from the AHE (ratio = 58.65) to HEV-ALF (ratio = 1.20) groups. The level of IFN-*γ* was associated with the outcome of HEV-ALF patients.

**Conclusions:**

HEV viral load was not associated with aggravation of AHE, and the HEV-ALF patients showed significant Th2 bias, which may be involved in the aggravation of AHE.

## 1. Introduction

Hepatitis E is an infectious disease of the digestive tract caused by hepatitis E virus (HEV) [1, 2]. It is mainly spread by the fecal–oral route, which is one of the main routes of transmission of hepatitis worldwide, and has become an important public health problem [3, 4]. Hepatitis E mainly occurs in developing countries and regions with backward sanitation conditions, which can spread infection [5]. In recent years, some developed countries, such as North America, Europe, and Japan, have also reported nonimported sporadic cases of hepatitis E [6]. There are four HEV genotypes, and those in China are mainly concentrated in types I and IV [7]. So far, only one serotype of HEV has been found. HEV can cause subclinical, acute, chronic, or severe infections in people of all ages and sexes [8, 9].

A large number of studies have confirmed that humoral and cellular immunity both play an important role in viral infection [10, 11]. In previous studies, Shen et al. [12] showed that CD8 of patients with hepatitis B virus-associated acute-on-chronic liver failure (HBV-ACLF) had obvious clonal expansion in the course of disease progression. The higher the degree of CD8 T cell clone expansion, the better the prognosis of HBV-ACLF patients. Han et al. [13] showed that patients with hepatitis C virus (HCV) infection had defective T cell function, and the direct effect of antiviral therapy improved the proliferation of HCV-specific CD8+ T cells. In a study by Shin et al. [14], providing nutritional education and food supplements to human immunodeficiency virus- (HIV-) infected women significantly increased weight and CD4+ T cells, and these interventions can be integrated into HIV care programs in low-income areas. Schlosse et al. [15] inoculated C57BL/6 mice, BALB/C nude mice, Wistar rats, and European rabbits with wild boar-derived HEV-3 strain, and monitored the replication and shedding of the virus and the humoral immune response to it. Remarkably, immunosuppressive dexamethasone treatment did not increase the susceptibility of rats to HEV infection. In rabbits, recombinant HEV-3 and rat HEV capsid protein induced a protective effect against HEV-3 infection. However, the involvement of cellular immunity in the development of HEV infection is rare. Although hepatitis E is self-limited, a growing number of cases of chronic infection or HEV-related liver failure have been reported [16, 17], especially in elderly people and pregnant women. It is important to investigate further the role of cellular immunity in hepatitis E development.

Hence, we conducted a correlation study in 34 patients with HEV infection, including 17 each with acute hepatitis E (AHE) or HEV-related acute liver failure (HEV-ALF). The study was carried out in response to the changes in T helper cell immune status and viral load in patients. To the best of our knowledge, this is the first study to characterize the immune mechanism of Th cells during HEV infection.

## 2. Materials and Methods

### 2.1. Study Population

We enrolled 34 patients with HEV infection, including 17 with AHE and 17 with HEV-ALF, who were referred to the First Affiliated Hospital, College of Medicine, Zhejiang University, between 10 September 2018 and 10 March 2019. The follow-up period ended in 9 March 2020. Another 17 healthy controls (HCs) were from the Health Examination Center of the First People's Hospital of Yancheng City. The present study was performed in accordance with the Declaration of Helsinki and was approved by the Ethics Committee of the First Affiliated Hospital, Zhejiang University (approval number: 2011013).

### 2.2. Definition and Clinical Classification of Cases

We defined AHE and HEV-ALF according to the King's College criteria as previously described [18]: AHE: (1) positive serum anti-HEV IgM and/or a greater than twofold increase in anti-HEV IgG titer and/or detectable HEV RNA and (2) combined with clinical presentation of acute viral hepatitis (e.g., elevated liver enzymes and/or jaundice and/or nonspecific symptoms, such as sudden onset of fever, vomiting, and nausea). HEV-ALF: (1) evidence of abnormal liver synthetic function (prothrombin activity ≤ 40% or international normalized ratio ≥ 1.5), jaundice, and hepatic atrophy over a 2-week period; (2) presence of stage 2 or 3 encephalopathy complicating end-stage disease manifestations; and (3) no chronic liver disease. Hepatitis A virus, HBV, HCV, and HIV infections were excluded from all enrolled patients and HCs, and HEV infection was also excluded from the HC group.

### 2.3. Data Collection

We collected all the data from the patients' medical records, including clinical baseline parameters, laboratory parameters, length of stay, and prognosis. The follow-up data were collected through medical records or by direct contact with the patients or their families, with death or liver transplantation as an endpoint.

### 2.4. HEV-Specific Antibody Detection

Diagnosis of hepatitis E was based on the presence of anti-HEV-IgM and IgG antibodies by ELISA, and only IgM- and IgG-positive cases were included. The presence of anti-HEV IgM and IgG antibodies was detected using commercially available HEV ELISA kits (Wantai, Beijing, China). The positive samples had optical density > 1.1.

### 2.5. HEV RNA Detection

HEV RNA was detected by internally controlled, quantitative real-time reverse transcription polymerase chain reaction (PCR), as previously described [18]. Total RNA was extracted and purified from serum using a viral nucleic acid purification kit (Aikang, Hangzhou, China). Nested PCR amplified a 348-nucleotide fragment of the HEV open reading frame 2, and the fragment of the HEV was sequenced to identify the genotype. The viral load of each sample was estimated by quantitative PCR, using a diagnostic kit for Hepatitis E Virus RNA (Aikang), with the following conditions: 30 min at 50°C, 2 min at 94°C, and 50 cycles of 30 s at 94°C, 30 s at 55°C, and 2 min 30 s at 68°C.

### 2.6. Isolation of Peripheral Blood Mononuclear Cells

At 4 days after initiation of detoxification treatment, peripheral blood samples (10 ml) were collected by venipuncture and placed in EDTA tubes. Peripheral blood mononuclear cells were isolated from fresh blood collected in K3 EDTA tubes using Ficoll density gradient centrifugation (GE Healthcare Life Sciences, Marlborough, MA, USA) for 30 min at 900 × *g*. Under a microscope (100x), cells were counted, and viability was always >95%, as determined by trypan blue exclusion (Sigma-Aldrich, St. Louis, MO, USA).

### 2.7. Immunophenotyping

A comprehensive panel of lymphocyte subsets was identified using multicolor flow cytometry. Peripheral blood mononuclear cells were washed in flow cytometry buffer (phosphate-buffered saline containing 1% fetal calf serum and 0.01% sodium azide) and then treated with flow cytometry blocking solution for 20 min. The cells were stained with combinations of anti-CD3, anti-CD8, anti-CD4, anti-CD56, and anti-CD16 monoclonal antibodies for 30 min at 4°C. Fluorescein isothiocyanate, phycoerythrin, and allophycocyanin were the fluorescent dyes, and all antibodies were purchased from BD Biosciences (San Jose, CA, USA). By using a FACS Canto II flow cytometer (BD Biosciences), at least 20,000 stained lymphocytes were identified by granularity and size. Data were analyzed by FlowJo version 7.2.5 software. CD3+ T cells were gated and displayed through histogram plots for other surface markers, and the percentage of cells showed the levels (mean ± SE).

### 2.8. Cytokine Measurements

ProcartaPlex Analyst 1.00 (eBioscience, San Diego, CA, USA) was used to determine the levels of plasma cytokines. By using a MILLIPLEX MAP Kit to analyze statistical data according to manufacturer's instructions, the value of samples was less than 0.2 pg/ml showing undetectable concentrations.

### 2.9. Statistical Analysis

All statistical analyses were performed with SPSS version 25 (IBM SPSS Statistics, Armonk, NY, USA). The continuous variables with normal distribution were expressed as the mean ± standarddeviation and tested with independent sample *t*-test. The variables with nonnormal distribution were expressed as median (IQR) and tested with the nonparameter test. The Mann–Whitney *U* test was used for group comparisons. The classified variables were tested by the chi-square test. A value of *P* < 0.05 was considered statistically significant.

## 3. Results

### 3.1. Characteristics of Study Subjects

The characteristics of the study subjects are summarized in Table 1. There was no significant difference among the three groups (*P* = 0.095), but the average age was 55.8 ± 7.3 years in the HEV-ALF group, which was significantly higher than in the AHE (45.7 ± 15.3 years) and HC (43.4 ± 14.9 years) groups. All enrolled 34 HEV patients were genotype 4. The average hospitalization time in the HEV-ALF group was 12 (8–23) days, which was significantly longer than in the AHE group (7 (5–11) days). Nine of the 17 patients in the HEV-ALF group recovered and eight died. All 17 patients in the HEV-ALF group had jaundice, eight (47.06%) had ascites, four (23.53%) had severity of hepatic encephalopathy, and 1 (5.88%) patient had hepatorenal syndrome. There were no pregnant women in the AHE and HEV-ALF groups.

### 3.2. Viral Load in the AHE and HEV-ALF Groups

**Figure 1:**
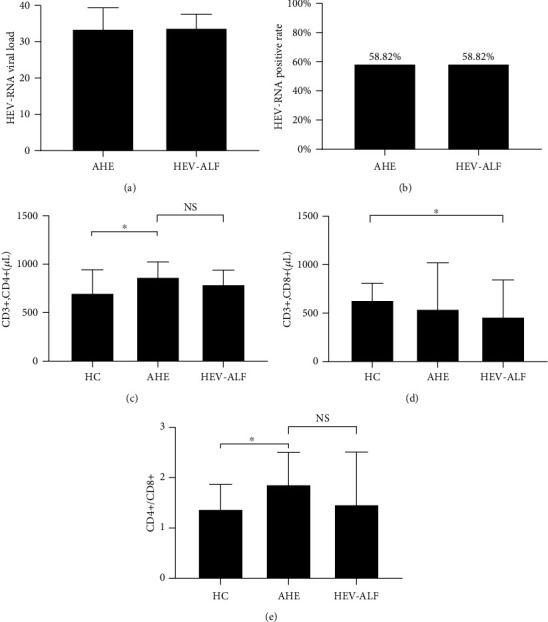
Viral load and lymphocyte levels in the AHE group and HEV-ALF group. (a, b) The viral load and positive rates of HEV-RNA in the AHE group and HEV-ALF group. (c–e) The CD3+CD4+, CD3+CD8+, and CD4+/CD8+ among the HC, AHE, and HEV-ALF groups.

Both the positive rates for HEV RNA in the AHE and HEV-ALF groups were 58.82%, and there was no significant difference in the viral load between the two groups (33.56 ± 5.81 vs. 33.85 ± 3.72; *P* > 0.05) (Figures 1(a) and 1(b)).

### 3.3. Th Lymphocytes and Th1/Th2 Cytokines

We measured the lymphocyte levels with anti-CD3, anti-CD4, and anti-CD8 antibodies in the HC, AHE, and HEV-ALF groups. The Th lymphocyte levels (CD3+, CD4+) in the AHE and HEV-ALF groups were significantly higher than in the HC group (both *P* < 0.05), but there was no significant difference between the AHE and HEV-ALF groups (*P* > 0.05). For cytotoxic T lymphocytes (CD3+, CD8+), there was no significant difference between the AHE and HC groups (*P* > 0.05), with similar conclusion between the HEV-ALF and AHE groups (*P* > 0.05), while cytotoxic T lymphocytes (CD3+, CD8+) in the HEV-ALF group were significantly lower than those in the HC group (*P* < 0.05) (Figures 1(c)–1(e)).

**Figure 2:**
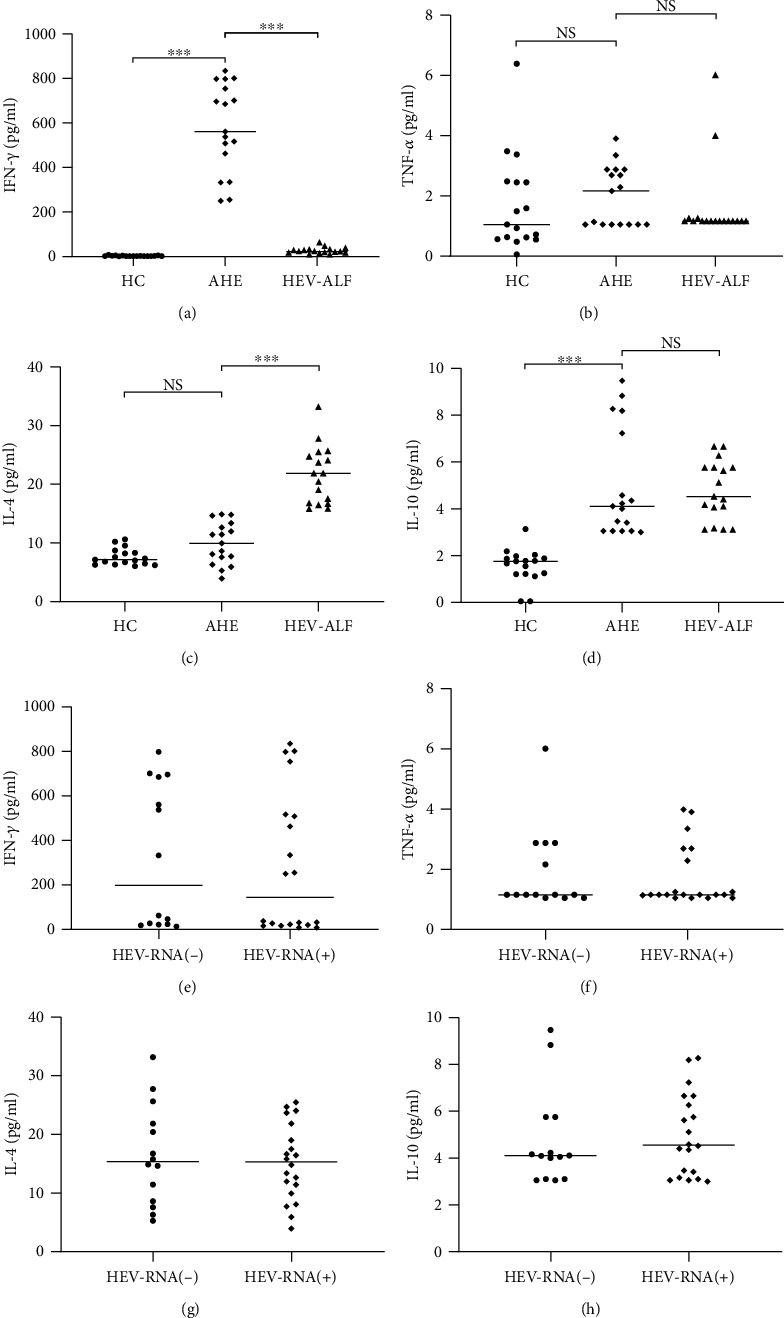
Compared with Th1/Th2 cytokines among the HC group, AHE group, and HEV-ALF group. (a, b) Th1 cytokines (IFN-*γ* and TNF-*α*) and (c, d) Th2 cytokines (IL-4 and IL-10) among the HC group, AHE group, and HEV-ALF group. (e, f) Th1 cytokines (IFN-*γ* and TNF-*α*) and (g, h) Th2 cytokines (IL-4 and IL-10) between the HEV RNA(+) group and HEV RNA(-) group.

Compared with Th1/T2 cytokines between the HC group and AHE group, both IFN-*γ* and IL-10 showed gradual upward trend from the HC group to the AHE (both *P* < 0.01), and there was no significant difference for TNF-*α* and IL-4 between the AHE and HC groups (both *P* > 0.05). Compared with Th1/Th2 cytokines between the AHE group and HEV-ALF group, IFN-*γ* showed a sharp downward trend from the AHE group to the HEV-ALF group (*P* < 0.01), while IL-4 showed gradual upward trend from the AHE group to the HEV-ALF group (*P* < 0.01), and there was no significant difference for TNF-*α* and IL-10 between the AHE and HEV-ALF groups (both *P* > 0.05; Figures 2(a)–2(d)).

In order to study the relationship between viral load and Th1/Th2 cytokines, we regrouped the 34 patients in the AHE and HEV-ALF groups according to whether HEV RNA was positive or not (HEV RNA(+) and HEV RNA(-) groups). There was no significant difference in Th1 (IFN-*γ* and TNF-*α*) and Th2 (IL-4 and IL-10) cytokines between the two groups (all *P* > 0.05; Figures 2(e)–2(h)).

### 3.4. Th1/Th2 Cytokine Production in the AHE and HEV-ALF Groups

To characterize the immune mechanism of Th cells during HEV infection, we compared Th1/Th2 (IFN-*γ*/IL-4) ratios among the groups (Table 2). Compared with the HC group, the AHE group showed Th1 bias (ratio = 58.65). Compared with the AHE group, there was a Th2 bias (ratio = 1.20) in the HEV-ALF group.

### 3.5. Correlation of ALT and TBIL and Severity of Hepatic Encephalopathy with Th2 Bias and IFN-*γ*

**Figure 3:**
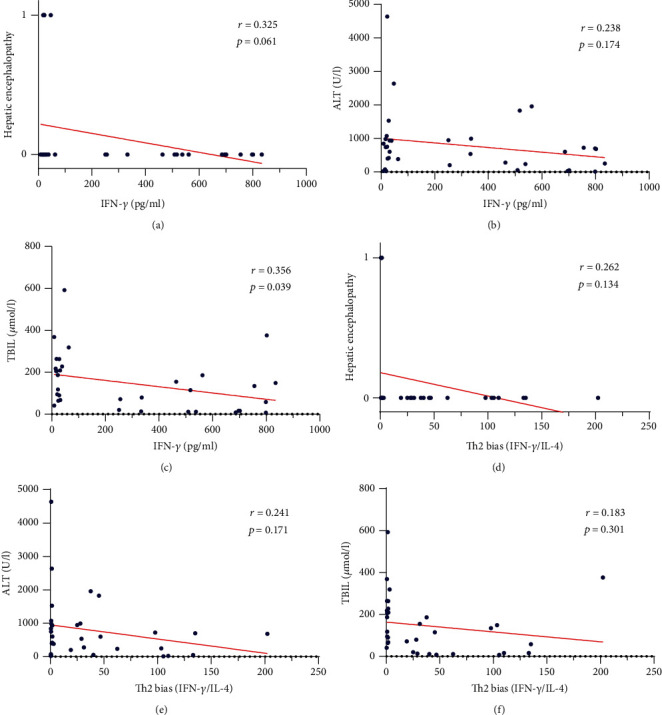
Correlation of ALT, TBIL, and severity of hepatic encephalopathy with Th2 bias and IFN-*γ*. (a–c) Correlation of severity of hepatic encephalopathy, ALT, and TBIL with the level of IFN-*γ*. (d–f) Correlation of severity of hepatic encephalopathy, ALT, and TBIL with Th2 bias.

We evaluated correlation of ALT and TBIL and severity of hepatic encephalopathy with Th2 bias and IFN-*γ* in the HEV-ALF group. The levels of severity of hepatic encephalopathy and ALT were weakly correlated with IFN-*γ* in the HEV-ALF groups (*r* = 0.325, *P* = 0.061; *r* = 0.238, *P* = 0.174; Figures 3(a) and 3(b)). The level of TBIL was strongly correlated with IFN-*γ* bias in the HEV-ALF group (*r* = 0.356, *P* = 0.039; Figure 3(c)). The level of ALT, severity of hepatic encephalopathy, and TBIL were weakly correlated with Th2 bias in the HEV-ALF groups (*r* = 0.262, *P* = 0.134; *r* = 0.241, *P* = 0.171; *r* = 0.183, *P* = 0.301; Figures 3(d)–3(f)).

### 3.6. Relationship between Th1/Th2 Cytokine Levels and Patient Outcome

We compared Th1/Th2 cytokine levels in 28 recovered patients and six patients who died. Only the level of IFN-*γ* in dead patients was significantly lower than in recovered patients (25.84 (16.63-30.23) vs. 399.03 (34.35-697.22); *P* = 0.006), while there were no significant differences for TNF-*α*, IL-4, and IL-10 between recovered and dead patients (all *P* > 0.05) (Table 3).

## 4. Discussion

The cellular immune response plays an important role in HEV infection [19, 20]. Although hepatitis E is an acute self-limited disease, chronic hepatitis E and hepatic failure caused by hepatitis E have been reported in recent years. A subset of immunosuppressed patients infected with HEV may develop chronic infection. The study by Ramdasi et al. [21] showed that HEV infection during pregnancy was highly correlated with the level of T regulatory cells and Th1 to Th2 shift. In cellular immunity, Th cells are normally in the precursor state. Under the influence of the virus, T cells differentiate and proliferate in different directions. Th1 cells mainly secrete cytokines such as IFN-*γ* and IL-2, which play an important role in antiviral and bacterial immune responses, while Th2 cells mainly secrete cytokines such as IL-4 and IL-10, which play a role in parasitic infection [22, 23].

In this study, we compared the changes in Th cell subsets by cell surface molecular staining. The numbers of Th lymphocyte levels (CD3+, CD4+) in the AHE group and HEV-ALF group were significantly higher than those in the HC group, but there was no significant difference between the AHE group and HEV-ALF group. Although the level of cytotoxic T lymphocytes (CD3+, CD8+) in the HEV-ALF group was significantly lower than that in the HC group, there was no significant change in the levels of Th2 cells between the AHE group and HEV-ALF group. We analyzed the changes in the levels of Th cell-related factors. The level of IFN-*γ* in the AHE group was significantly increased. Although IL-10 also showed a significant increase, we considered that Th1 cells were involved in HEV infection and virus clearance. It should be noted that IFN-*γ* decreased sharply from AHE to HEV-ALF. At the same time, this process is accompanied by increased level of IL-4. Significant Th2 bias was observed from AHE to HEV-ALF. We inferred that hepatocyte damage was aggravated due to the persistent imbalance of immune status in the body. This result is consistent with Ravi and Arankalle [24] and Majumdar et al. [25].

We compared the HEV positive rates and viral loads in the AHE and HEV-ALF groups. There was no significant difference in the HEV positive rate or viral load between the two groups, suggesting that HEV viral load was not associated with disease severity. There was no significant difference in Th1/Th2 cytokines between the HEV or HEV-ALF groups. All the above results may indicate that the HEV viral load has little effect on Th1/Th2 cytokines, but it may also be related to the positive feedback of Th1/Th2 cell proliferation on immunity. Sex and age should be excluded, and the results were verified in more samples.

Both IFN-*γ* and Th1 bias were negatively correlated with ALT, and severity of hepatic encephalopathy, especially for TBIL. Although HEV was cleared in many patients, ALT and bilirubin levels indicated further hepatocyte damage, and we speculate that hepatocyte damage may not be directly caused by HEV, but rather by Th2 bias caused by restraint of Th1 cells. HEV-ALF patients had a high mortality rate. Hence, we evaluated the relationship between Th1/Th2 cytokine levels and outcome of HEV-ALF patients, and only the level of IFN-*γ* was associated with outcome.

Cytokines are important immune messenger molecules, which are secreted in the blood, and they are affected by a variety of viruses and bacteria. The study cohort excluded interference by other infectious diseases, tumors, age, sex, and other factors. Our research also considered the effect of lifestyle, environment, and other factors. None of these could completely exclude the influence of other organs and factors, which needs to be confirmed by a large multicenter study.

In summary, HEV viral load was not associated with aggravation of AHE. The IFN-*γ* levels showed a gradual upward trend from the HC group to the AHE group, while it showed a sharp downward trend from the AHE group to the HEV-ALF group and the HEV-ALF patients showed significant Th2 bias. The level of IFN-*γ* was associated with the outcome of HEV-ALF patients. We consider that Th2 bias may be involved in the aggravation of AHE.

## Figures and Tables

**Table 1:** Characteristics of study subjects.

	HC group (*n* = 17)	AHE group (*n* = 17)	HEV-ALF group (*n* = 17)	*P* value
Age (y)	43.4 ± 14.9	45.7 ± 15.3	55.8 ± 7.3	0.018
Gender (F/M)	9/8	7/10	3/14	0.095
Pregnant woman	0	0	0	—
Fever	0	4 (23.53)	7 (41.18)	0.014
Jaundice	0	15 (88.24)	17 (100.00)	≤0.001
Nausea/vomit	0	5 (29.41)	9 (52.94)	0.002
Severity of hepatic encephalopathy	0	0	4 (23.53)	0.013
Hepatorenal syndrome	0	2 (11.76)	1 (5.88)	0.352
Ascites	0	2 (11.76)	8 (47.06)	≤0.001
IgM(+)	0	17 (100.00)	17 (100.00)	≤0.001
HEV-RNA (IU/ml)	0	34.84 (29.18-38.73)	33.44 (30.55-37.74)	≤0.001
ALT (U/l)	18 (9-31)	535 (128-834)	750 (392-1027)	≤0.001
TBIL (*μ*mol/l)	12.4 (8.4-14.2)	58.0 (12.2-141.6)	206.6 (92.0-263.4)	≤0.001
Length of stay (day)	0	7 (5-11)	12 (8-23)	≤0.001
Mortality rate	0	0	8 (47.06)	≤0.001

The *P* value was for the difference among the three groups.

**Table 2:** Th1/Th2 (IFN-*γ*/IL4) ratios in the AHE group and HEV-ALF group.

Categories	IFN-*γ* (pg/ml)	IL-4 (pg/ml)	IFN-*γ*/IL-4 ratio
HC group	2.94	7.63	0.39
AHE group	578.24	9.86	58.65
HEV-ALF group	25.78	21.57	1.20

**Table 3:** Relationship between Th1/Th2 cytokine levels and outcome of patients.

Th1/Th2 cytokines	Recovered patients (*N* = 26)	Dead patients(*N* = 8)	*P* value
IFN-*γ*	399.03 (34.35-697.22)	25.84 (16.63-30.23)	0.006
TNF-*α*	1.16 (0.05-2.88)	1.16 (1.16-0.25)	0.205
IL-4	12.31 (8.48-23.78)	16.70 (15.98-18.64)	0.167
IL-10	4.23 (3.78-5.00)	5.36 (3.42-5.75)	0.393

## Data Availability

All data relevant to the study are included in the article.

## Abbreviations

HE: Hepatitis E
HEV: Hepatitis E virus
AHE: Acute hepatitis E
HEV-ALF: HEV-related acute liver failure
HC: Healthy controls
ELISA: Enzyme-linked immunosorbent assay
Th: Helper T cell
ALT: Alanine aminotransferase
TBIL: Total bilirubin.
